# The Number of Subgrain Boundaries in the Airfoils of Heat-Treated Single-Crystalline Turbine Blades

**DOI:** 10.3390/ma14010008

**Published:** 2020-12-22

**Authors:** Jacek Krawczyk, Włodzimierz Bogdanowicz, Jan Sieniawski

**Affiliations:** 1 Institute of Materials Engineering, University of Silesia in Katowice, 75 Pułku Piechoty 1a St., 41-500 Chorzów, Poland; wlodzimierz.bogdanowicz@us.edu.pl; 2 Department of Materials Science, Rzeszów University of Technology, Wincentego Pola 2 St., 35-959 Rzeszów, Poland; jansien@prz.edu.pl

**Keywords:** superalloys, low-angle boundaries, X-ray topography, turbine blades, crystal growth

## Abstract

In the present study, the dendrites deflection mechanism from the mold walls were subjected to verification regarding its heat-treated turbine rotor blades. The number of macroscopic low-angle boundaries created on the cross-section of the blades’ airfoil near the tip was experimentally determined and compared to the number of low-angle boundaries calculated from a model based on the dendrites deflection mechanism. Based on the Laue patterns and geometrical parameters of airfoils, the number of low-angle boundaries occurring at the upper part of the blades airfoil after heat treatment was calculated. This number for the analyzed group of blades ranged from 5 to 9.

## 1. Introduction

The turbine components of aircraft engines are currently most often produced using the CMSX-4 superalloy. The single-crystalline parts made using the CMSX-4 superalloy possess high strength properties, even at high temperatures, which is especially important for turbine rotor blades, as these types of blades are exposed to harsh working conditions [1,2,3,4,5]. The single-crystalline rotor turbine blades, which have a very complex shape, are usually produced by using the Bridgman technique [6,7,8,9,10,11,12]. The blades are obtained through directional crystallization using the temperature and withdrawal parameters that allow the formation of an array of dendrites nearly parallel to the blade axis, with the crystal orientation of each dendrite being parallel to the [001] axis. The dendrites and interdendritic areas that are formed during the Bridgman process mainly consist of the Ni-based γ primary solid solution and the Ni_3_Al-based γ’ secondary solid solution [13,14,15,16]. Due to similarity between the structure of both phases and the possibility of obtaining a clear X-ray diffraction pattern, the blades can be recognized as single-crystalline blades [17,18,19].

The production of the rotor blades includes subjecting them to the heat treatment process to increase chemical and microstructural homogeneity and to obtain a large amount of the γ’ phase, as well as to decrease the crystal orientation inhomogeneity through elimination of the low-angle boundaries (LABs) created during crystallization [20,21,22,23]. However, it has been stated that not all macroscopic LABs are eliminated by the heat treatment but also that new extra LABs may be created as a result of this treatment. All of the LABs decrease the strength of the rotor blades [24,25,26].

An airfoil is generally a thin-walled fragment of a blade and has the least durability of all parts of a blade due to its low cross-section and the large complex loads that are acting on it during its operation [27]. Therefore, analysis of the number of defects such as LABs in the treated blades airfoils is extremely important. The number of the LABs with respect to the stretching direction has a strong effect on the creep life of the blades. The LABs also affect the adjacent dislocation density, which is also related to the misorientation angle. It is widely accepted that the boundaries with a misorientation of above 1° have a significant influence on the mechanical properties of the LABs. In a thin-walled airfoil, the interaction of the dendrites with the mold walls may occur more frequently than in the root; therefore, the LABs can be formed more often [28].

During directional crystallization, the dendrites grow directly toward the [001]-type direction, which is only close to the blade axis Z. Therefore, even if the mold walls are parallel to the Z, this means the dendrites can contact them. Additionally, the inclination of the airfoil surfaces in relation to the Z axis of the rotor blade makes the growing dendrites contact the mold walls and interact with them. The character of interactions may depend on the angle between the axis of the dendrite primary arms and the mold surface [28]. For lower angles, the dendrite primary arms may slightly change their growth direction near the surface, thereby creating areas of internal stress, and then stop. The higher angles prevent a further growth of the primary dendrite arms without a change in the growth direction. In both cases, further growth of a dendrite takes place due to the secondary arms that are perpendicular to the primary arms [29]. Both mechanisms can lead to the formation of low-angle boundaries. In the former case, these boundaries may be formed after heat treatment, for example through the process of dislocation polygonization, while in the latter case the boundaries may be formed directly during crystallization. These mechanisms of dendrite interaction with the mold walls were proposed for the first time in Refs. [28,30] and were referred to as the deflection of dendrites on the mold surfaces.

The aim of the research presented here was to check the assumption that in heat-treated single-crystalline blades made of a Ni-based superalloy, each type of dendrite deflection on the mold surface creates LABs. The assessment was performed by using experimental determination of the LABs number in thin-walled blade airfoils where the probability of dendrites interaction with the mold walls is high, and a comparison with the number of LABs could be determined by using a model based on the aforementioned assumption. The kinetics of the dendrites growth may affect the deflection mechanisms; therefore, it was decided to examine blades airfoils that were obtained at different withdrawal rates in the range of 2–5 mm/min, including the most commonly used rate of 3 mm/min.

## 2. Material and Methods

**Figure 1:**
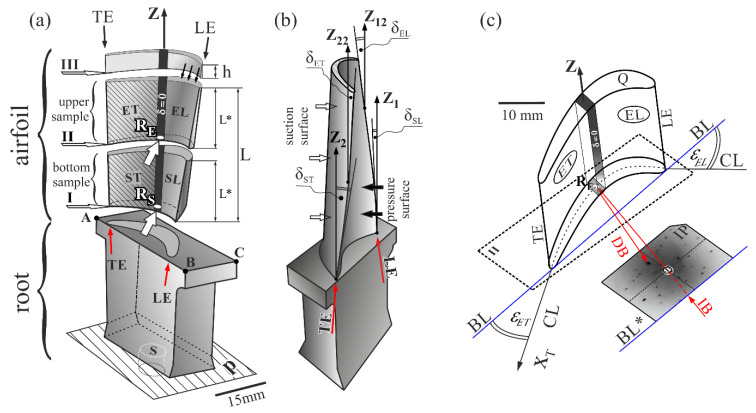
(**a**) Illustration of a turbine blade with a scheme of cross-sections I, II and III and the location of the ET, ST, EL and SL areas: (**b**) description of airfoil surface inclinations relative to the axis Z of a blade near the leading edge (LE) and trailing edge (TE); (**c**) a scheme for obtaining the Laue pattern for the upper sample of an airfoil with an exemplary Lauegram. CL—camber line, BL—base line of cross-section II of an airfoil, δ_SL_, δ_EL_, δ_ST_ and δ_ET_—angles of inclination of LE and TE relative to the axis Z, ε_EL_ and ε_ET_—angles of airfoil camber, describing the rotation of airfoil surfaces to the BL, IB—incident X-ray beam, DB—diffracted X-ray beam, IP—image plate in which the Laue pattern was obtained. Z is perpendicular to the base plane P; Z_1_, Z_12_, Z_2_ and Z_12_ are parallel to the Z.

The Bridgman technique was used for production of two series of single-crystalline turbine rotor blades. The as-cast blades made of CMSX-4 Ni-based superalloy were heat treated and then analyzed. Each series consisted of four blades obtained at different withdrawal rates. The directional crystallization with the use of a spiral selector (S, Figure 1a) and heat treatment process was performed in the Research and Development Laboratory for Aerospace Materials at the Rzeszów University of Technology, Rzeszów, Poland using industrial ALD Vacuum Technologies furnaces. In each series, four crystallization processes were carried out at four different withdrawal rates: 2, 3, 4 and 5 mm/min. The heat treatment, performed by using several steps, consisted of convection heating to 950 °C (in Ar or He), radiation heating to 1350 °C (in vacuum), solution annealing and aging. The temperature-time settings for annealing were: 1277 °C/4 h → 1287 °C/2 h → 1296 °C/3 h → 1304 °C/3 h → 1313 °C/2 h → 1316 °C/5 h → gas furnace quench; for aging, the settings were: 1140 °C/6 h (step 1) and 871 °C/20 h (step 2).

X-ray diffraction topography and Laue back-reflection diffraction were used to analyze the LABs and define the crystal orientation of airfoil samples. The Laue method is the basic method for defining the crystal orientation which many automatic programs and indexing systems are also based on [31]. The Panalytical X-ray system (Alamelo, The Netherlands) equipped with a microfocus tube (with a quasi-point source of 40 × 40 µm^2^) emitting characteristic CuKα divergent beam radiation was used for topography studies. The anode current of 0.3 mA and an anode-cathode voltage of 30 kV were applied. The topograms of the 113 reflection, which is the reflection with the highest intensity for the analyzed sample surface (surfaces of the samples are parallel to the (001) crystal plane), were recorded on the AGFA Structurix D7 X-ray film with a grain size of 7 μm. The oscillations of the coupled sample and film were applied during exposure of the topograms. The source-to-sample distance was 25 mm and the sample-to-film distance was 10 mm. The details of the experiment are presented in Appendix A. The Laue patterns were obtained on the image plates using the X-ray diffractometer of the RIGAKU/EFG XRT-100CCM system provided by EFG Freiberg Instruments (Freiberg, Germany). The accuracy of the angle measurement in the Laue diffraction method was determined by using the spot size and the precision of the sample positioning in the diffractometer holder. The reference plane of the goniometer allowed us to set the sample in the holder with a mean error of about 0.3°. To determine the orientation measurement error related to the size and shape of the Laue spots, a circular envelope of each spot was outlined and the center of the envelope was found. The longest distance from the spot center to the envelope for the Laue pattern was the mean error of 0.5°.

There are several methods that can be used to visualize low-angle boundaries in single crystals. They mainly differ in the type of X-ray source, shape and width of the incident beam, spatial and angular resolution. Table 1 presents some parameters of these X-ray topography methods and their application for different materials. The methods allowing for higher limit resolution with the use of conventional X-ray sources, e.g., the Berg-Barrett or Lang methods, use a highly collimated narrow incident beam. There are two drawbacks to using such methods when examining large engineering elements: the area covered by the X-ray beam is relatively small and may not cover the entire sample; additionally, some areas of the sample with a higher misorientation angle (e.g., several arc degrees) may not be visible on the topogram due to a failure to meet the Bragg condition. This made it impossible to visualize all LABs that may be present in the single-crystalline casts made of the superalloys on the one topogram. Such casts, obtained by using the Bridgman method, consist of a set of almost parallel dendrites and their groups (called subgrains) with a fairly large dispersion of the misorientation angle, including from arc minutes to angular degrees [32]. The X-ray topography with a divergent width beam and oscillation of the coupled sample and film that was applied in this study seemed to meet all of the relevant requirements and was suitable for the visualization of all LABs in the relevant casts.

The rotor blades casts are divided into two main parts—bulk root and fine airfoil (Figure 1a). Three cross-sections I, II and III of the airfoils were made for each analyzed blade. The first section (I) was localized near the platform ABC of the root (Figure 1a). The third cross-section (III) was cut off of the airfoil’s tip part with a height h = 3 mm, and the second cross-section (II) divided the remaining fragment of the airfoil with the height L into two parts named the bottom sample and the upper sample. The bottom and the upper samples had the same height L* = L/2. The tip parts of the airfoils were not studied because they contained high internal stresses that made it impossible to create clear diffraction images. The airfoils of rotor blades are bounded by two surfaces, the suction and pressure surface, which are indicated in Figure 1b by white and black arrows, respectively. Both surfaces are twisted around blade’ axis Z like a clockwise screw. The twist can be defined by continuous rotation of the chord line called the base line (BL) (Figure 1c) at each transverse section along the axis Z. In addition to this rotation, changes in the cambers of the airfoil may occur, which are related to changes in angles of the airfoil surfaces’ rotation to the BL, which can be approximately described by the angles ε, marked for example in Figure 1c as ε_ET_ and ε_EL_ for the upper airfoil samples. The angles ε may be defined as the angles of rotation of camber line fragments located near the leading edge (LE) and the trailing edge (TE).

The values of the angles ε_EL_ and ε_ET_ are different for the cross-sections I and II because the rotation of airfoil surfaces to the BL change continuously along the axis Z (Figure 1a). The suction and pressure surfaces are inclined towards the axis Z. These inclinations lead in turn to inclinations of the LE and TE to the Z axis (Figure 1b). The inclination angles δ of the LE and TE are different to those of the angles ε, and the characteristics of the δ angles’ changes along the Z axis are also different. The angle δ for the LE increases along the axis Z (δ_EL_ > δ_SL_, Figure 1b) and decreases for the TE (δ_ET_ < δ_ST_, Figure 1b). There is a narrow area in the central part of the airfoil, marked in Figure 1a,c in black, for which both the suction and pressure surfaces are parallel to the axis Z. The inclination angle of the area is δ = 0 along the entire height of the airfoil from its beginning near the root to its tip. The geometric dimensions, including the length and thickness of the airfoil and the angles of its edges, were obtained using a 3D scanner. The measurement accuracy was 0.2° for the angles and 0.1 mm for the length dimensions.

In order to experimentally determine the number of LABs, X-ray diffraction topograms were obtained from the upper sample surfaces marked in Figure 1a by thin black downward arrows. To obtain the topograms, the samples were oriented using an additional Laue back-reflection diffraction from the surface Q of section III (Figure 1a,c). The macroscopic LABs are planar defects and their surfaces in airfoils are approximately parallel to the axis Z of the blade [28]. Therefore, it can be assumed that the LABs created during crystallization in the lower part of the airfoil with any distance from the platform ABC would be extended along the axis Z—the axis which indicates the direction of the crystallization process—and would pass through the upper parts of the airfoil. Therefore, in cross-section III near the tip of the airfoil, all of the low-angle boundaries created during crystallization of the airfoil would appear.

## 3. Results and Discussion

**Figure 2:**
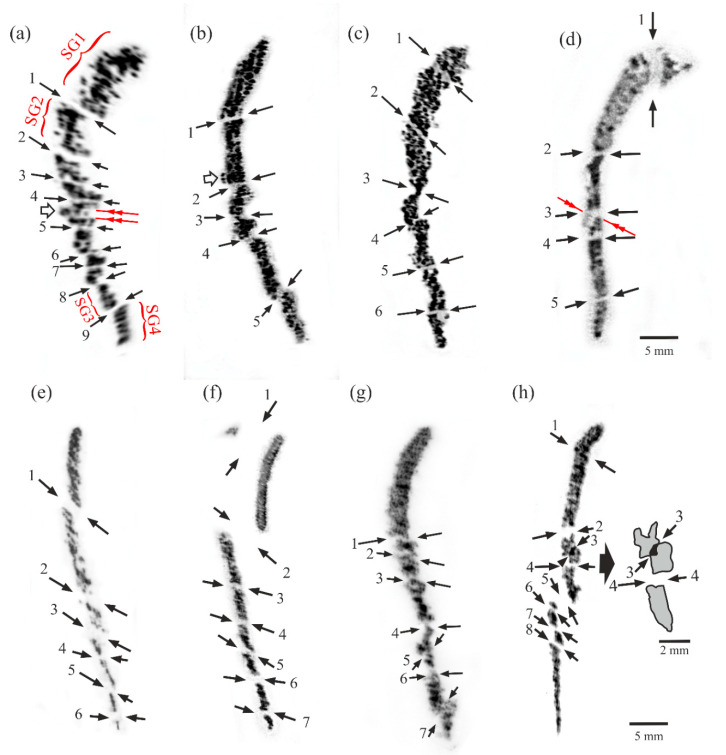
X-ray topograms obtained from micro-section III of an airfoil of I-series (**a**–**d**) and II-series (**e**–**h**) obtained at a withdrawal rate of 2 mm/min (**a**,**e**), 3 mm/min (**b**,**f**), 4 mm/min (**c**,**g**) and 5 mm/min (**d**,**h**). Reflection 113. CuKα radiation.

Figure 2 shows X-ray topograms obtained for airfoils of two series of blades obtained at the withdrawal rates of 2 mm/min, 3 mm/min, 4 mm/min and 5 mm/min each. The presented topograms were obtained from the surface Q of the upper sample located near the tip of the airfoil, where the crystallization of the blades was coming to an end. The topograms obtained from the cross-section III contain images of all of the LABs created during the passage of the crystallization front along the axis Z across the entire length of the blade airfoil, as well as images of LABs that were inherited by the airfoil from the root [28]. The topograms consist of the groups of contrast stripes or/and spots marked for example as SG1, SG2, SG3 and SG4 in Figure 2a. The individual contrast spots and stripes visualize the separate dendrites while the groups indicate the individual subgrains (which are SG type subgrains—see Figure 2 for further details). The mutual shift of the images of neighboring dendrites or the images of the neighboring subgrains allows for determination of the angle of their mutual misorientation, which also describes the disorientation angle of the LABs. The mutual misorientation angle calculation method is described in Ref. [34]. The misorientation angle between the single dendrites was found to be up to 0.3°, and it was higher between groups of dendrites. This criterion allowed us to identify the subgrain in nickel-based dendritic superalloys.

There were visible bright bands with a low-contrast (or a lack of contrast) between the images of subgrains. The bands represent LABs between subgrains. The areas of adjacent subgrains in the topograms were spaced and/or shifted relative to each other. Sometimes the low-angle boundaries were visualized in the topograms by using increased contrast, as presented in Figure 2h—LAB no.3. The reason for the above is that the crystal lattices (diffraction planes) of certain neighboring subgrains were inclined toward each other in such a way that their images in the topograms partially overlapped.

The LABs with misorientation angles of above 0.3° are marked by the arrows in Figure 2 and numbered, which allows determination of their number N. The error of the misorientation angle measurement depended on many factors related to the material of the sample and the instrument error. The mean orientation error for the presented results was about 8 arc minutes. The selection of the LAB in the topograms was based on the criteria described in detail later and related to the mechanism of LABs creation.

**Figure 3:**
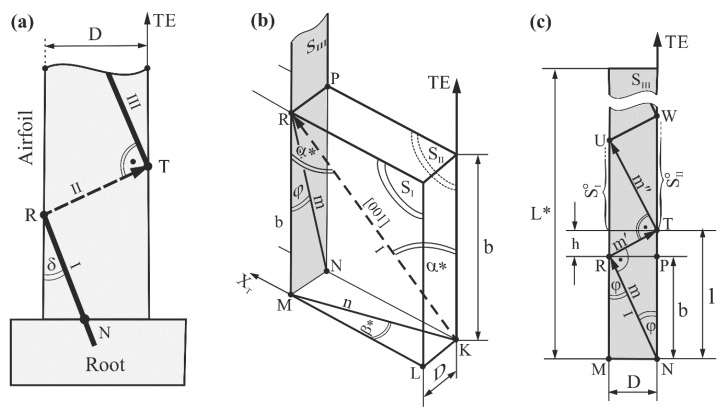
Schemes of dendrite deflection from the mold walls’ surfaces parallel to the TE in the ET fragment (**a**) of the blades airfoil. The δ, φ, α* and β* angles are enlarged for figure clarity. Section m in (**b**,**c**) is the projection of the primary arm direction parallel to the [001] on the plane defined by the points M, N, P and R which is perpendicular to the axis X_T_ of the CL (**c**), and sections m’ and m” in Figure 3c are the projections of secondary and tertiary arms deflected from the fragments S°_I_ and S°_II_ of mold walls planes of the ET area.

The boundaries creation may be related to the mechanism of the growing dendrites deflection from the surface of the casting mold, which was first proposed in Ref. [28]. This mechanism was based on stopping the primary arm growth on the mold wall (at the point R, see Figure 3a) and the continuation of crystallization by the secondary arms, and then (at the point T, see Figure 3a) by the tertiary dendrite arms. Because the secondary dendrite arms were arranged in the arrays connecting the sucking and pressure surfaces of the airfoil, their image (and also the image of the LABs) was visible along the entire width of the topogram. Such a growth path occurs when the angle δ between the primary arms and the mold wall is higher than the critical one [30]. At a very low δ angle (Figure 3a) below the arc minute, the dendrites do not deflect on the mold wall—in this case, the dendrites bend and continue to grow parallel to the TE axis, while LABs are not formed either during the dendrite growth from the melt or after the heat treatment. When the angle δ is higher but remains lower than the critical level, then the primary dendrite arm may bend before deflection, which leads to creation of an area of internal stress where extra LABs can also be formed after heat treatment [36]. The surfaces of these LABs are approximately parallel to the blade axis Z [28,30]. Therefore, in the airfoil cross-section III (Figure 1a), all LABs—which formed as a result of deflections that occurred along the entire airfoil height L (Figure 1a)—appeared. It follows that to theoretically calculate the number N of the low-angle boundaries visualized on the cross-section III, it was necessary to calculate the number of acts of dendrites deflection from the surface of the mold walls.

The number of deflections is particularly important for fine airfoils. Although the angle δ is usually low (less than 12–15°) because the airfoil dimension D is also low (Figure 3a), the number of deflections is fairly high as a result. The mechanisms for LABs creation in the bulk root connected with the selector provide different results. The main reason for this is a fast, unsteady lateral growth of dendrites near the selector-root connection surface [37]. Usually, two LABs are formed in the root, which are inherited by the airfoil [28]. These macroscopic LABs created in the root are tens of millimeters long. The LABs that are inherited by the airfoil pass through its entire cross-section. Taking the aforementioned mechanisms into account, the criteria used to indicate the LABs image in the topograms can be described as follows: the decreased or increased contrast bands representing the LABs must cross the entire width of the topogram; these contrast bands must be formed between the other contrast bands representing the groups of the dendrites; and the contrast bands representing the dendrite group (subgrains) must be shifted integrally relative to the adjacent bands representing the other group. Typically, the shift of the contrast bands representing subgrains is higher than the shift between the contrast spots and/or the stripes representing the individual dendrite, i.e., a shift greater than 0.3°. The error in determining the misorientation angle is very important when calculating the number of LABs. The LABs may be created as a result of deflection; therefore, the internal stresses may appear, causing blurriness or/and a bend of the contrast line in the topograms (e.g., SG3, Figure 2c). A large misorientation of the single dendrite can also occur, which is visualized in the topogram by a greater shift between the contrast bands visualizing the neighboring dendrites (above LAB no.6 in Figure 2h). As a result of these phenomena, incorrect LAB identification may occur, resulting in inaccurate counting.

The resolution of the method for determining the number of LABs is related to the resolution of the X-ray topography, but it is not crucial in this case. The aim of the experiment was to determine the number of macroscopic LABs formed as a result of the dendrites deflection mechanism using recorded topograms. Theoretically, for almost perfect single-crystals, the resolution of the applied X-ray topography method is several arcmin [34]. However, for the dendritic single-crystalline nickel-based superalloys of the CMSX-4 type, the resolution ranges from a dozen to several dozen arcmin. The outcome depends on the existing internal stresses, the X-ray background level and the arrangement of the diffraction planes in relation to the analyzed surface. For the CMSX-4 containing several alloying elements, it also depends on the heterogeneity of the spatial distribution of these elements. Although the resolution of the applied method is lower than that of the Berg-Barrett or Lang method, it is still useful for visualizing all sample areas with high misorientation angle ranges from a dozen arcmin to several angular degrees on the one topogram [32]. Additionally, the divergence of the X-ray beam allowed us to obtain the topograms from large sample surfaces—even up to approximately 10 cm^2^. This is very important for testing engineering products such as turbine blades.

The linear resolution of the applied method was in the order of 100 µm for almost perfect single-crystals. The thickness of the dendrites in the analyzed samples of superalloys obtained by using the Bridgman technique with the withdrawal rate from the high temperature zone of 2–5 mm/min ranged from 300 µm to 100 µm [1], so in this case the images of all dendrites could be seen on the topograms. As the images of the dendrites were visible in the topograms (Figure 2) in the form of stripes or spots, the linear resolution limit ranged from 100–300 µm. In the presented research, it was necessary to visualize all possible existing LABs with a misorientation angle ranging from several arcmins to several degrees of arc. It was not necessary to increase the resolution to arcsec, which could have been achieved by using the Berg-Barrett method. The scheme of the primary dendrite arm arrangement, for example in the fragment ET with the thickness D and height L* (Figure 3b,c), may be used to calculate the number of dendrite deflections. It can be correctly assumed that a dendrite grows directly toward the direction [001], as is commonly believed to occur [38].

S_I_ and S_II_ in Figure 3b are the side walls of the blade airfoil (and the casting mold) parallel to the TE (Figure 1c). The direction [001] is the direction of the primary dendrite arm that reaches the point R and then deflects. The unit vector KR indicates the direction of the dendrite growth. Then the dendrite growth was continued by the secondary arm up to the point T (Figure 3c). The tertiary arms growth began at the point T and reached the point U where subsequent deflection occurred. In order to simplify considerations, it was assumed that the angle between the subsequent rows of dendrite arms is a right angle (Figure 3c). Since the distance D is small, and the secondary dendrite arm is almost perpendicular to the mold walls, the distance h (Figure 3b) was small in comparison to b, therefore it could be assumed that l ≈ b. Using the above assumptions, the Equation (1) that allowed us to calculate the number of deflections N was obtained (see Appendix B):

(1) $$N=\frac{L*}{D}\times \tan\alpha *\times \sin\beta *$$

where α* is the angle between the direction [001] and the TE, and β* is the angle between the projection of the direction [001] on a certain plane (the plane is perpendicular to TE) and the axis X_T_ (the axis is parallel to the fragment CL of the airfoil area ET) (Figure 1c). The angles could be determined using the Laue patterns of the airfoil cross-sections. The Laue patterns were obtained by arranging the baseline BL* of the image plate parallel to the baseline BL of the airfoil cross-section (Figure 1c). The primary X-ray beam was directed at the airfoil region with δ = 0. The Laue patterns were obtained from the points R_S_ and R_E_ (Figure 1a) of the bottom and the upper samples of each blade airfoil, respectively.

An airfoil can be divided into five areas: one area with the angle δ = 0, the two areas SL and EL (Figure 1a) (their inclination to the axis Z can be referred to as the inclination of the LE relative to Z) as well as the two areas ST and ET (Figure 1a) (their inclination can be referred to as the inclination of the TE). The area with δ = 0 is the middle area and was not considered because it is relatively narrow (Figure 1a). The areas SL and ST of the bottom sample, as well as the areas EL and ET of the upper sample, are rotated around the BL of the bottom and the upper samples by the angles ε and are inclined to the blade axis Z by their respective angles δ.

**Figure 4:**
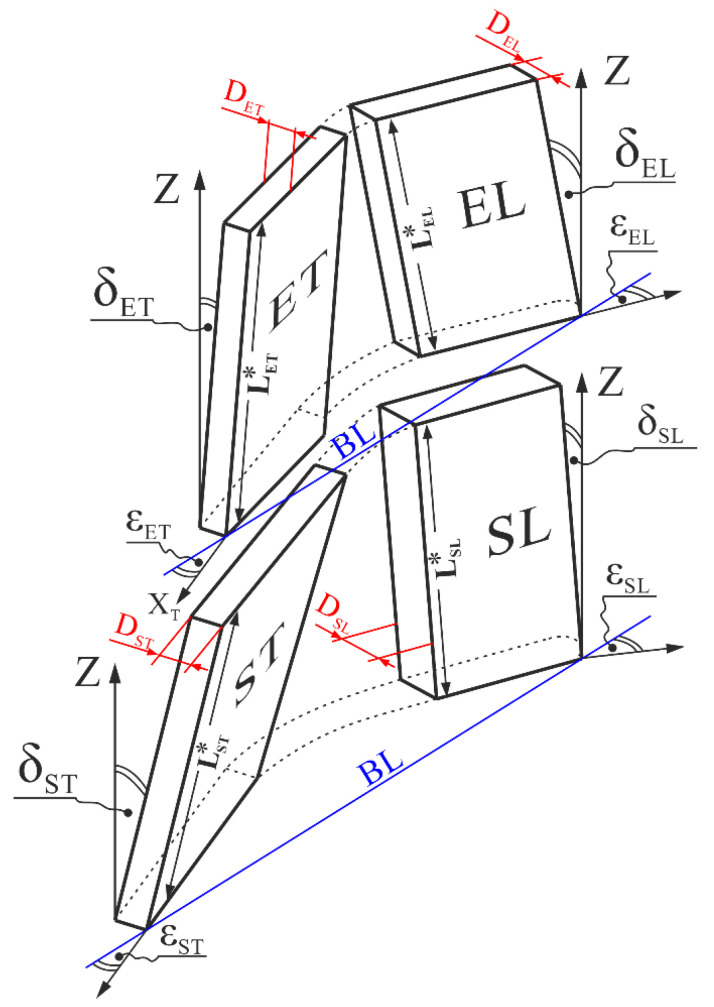
Model scheme of the areas SL and ST of the bottom sample, as well as the areas EL and ET of the upper sample of the blade airfoil with the characteristic parameters of the blades geometry.

Figure 4 shows a model fragmentation and arrangement of the four airfoil areas with their geometrical parameters. The areas were modeled as plates with parallel surfaces of the average thickness D and height L*. The values of the angles δ and ε, as well as the value of L* = L/2 (Figure 1a) for concerned areas are presented in Table 2.

To determine the number of deflections N of each of the areas SL ST, EL and ET using the Equation (1), it was necessary to experimentally determine the angles α* and β* of these areas.

**Figure 5:**
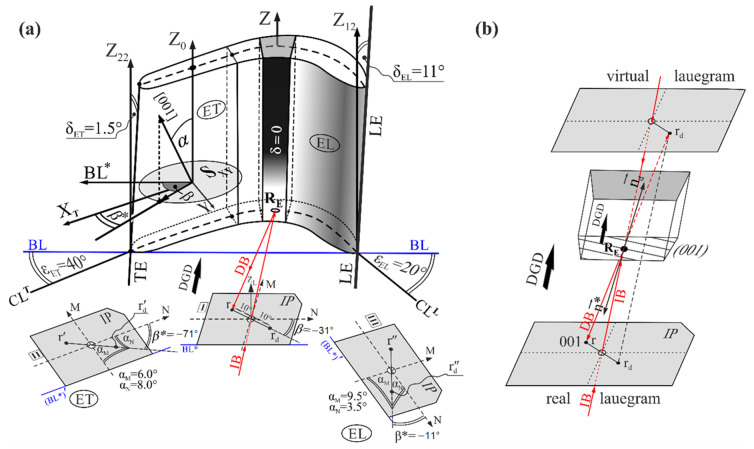
Exemplary illustration of the areas EL and ET of the upper sample of an airfoil obtained at the withdrawal rate of 5 mm/min, with geometric description of angles α, β and β* (**a**) and a diagram explaining how to determine the position of the spot r_d_ on the lauegram through inversion of the spot r (**b**). The plane S_XY_ is parallel to the plane p and cross-sections I, II and III in Figure 1a, while the BL* of the lauegram is parallel to the BL of an airfoil cross-section. IP—image plate, IB and DB—incident and diffracted X-ray beam, DGD—dendrite growth direction. $\vec{n*}$ and $\vec{n_{d}}$—unit vectors which proceed in a normal direction towards the diffraction plane (001) with the direction corresponding to the diffracted beam and the dendrite growth direction. Z_0_, Z_12_ and Z_22_ are parallel to the Z axis of the blade.

Figure 5 shows a scheme of the areas EL and ET of the upper sample, as well as arrangement of the image plate in which the Laue back-reflection pattern was recorded. During the experiment, both the sample surface and the image plate were positioned vertically, and the X-ray beam was positioned horizontally. The diffracted beam (Figure 5b) was directed in the opposite direction to the dendrites growth direction. The crystallographic orientation—which determines the direction of the growing dendrite—is represented by the spot r_d_ on the real lauegram. The spot is obtained by using inversion of the 001 reflection (r spot) relative to the center of the lauegram. The position of the Laue spot r is determined by using the vector $\vec{n*}$, which proceeds in a normal direction towards the diffraction plane (001), and its direction corresponds to the diffracted beam. The direction of the vector $\vec{n_{d}}$, which also proceeds in a normal direction towards the diffraction plane (001), corresponds to the direction of the growing dendrites. The extension of the diffracted beam up to the intersection with the created virtual lauegram (Figure 5b) determines the location of the spot r_d_. To determine the location of the r_d_ spot, a virtual lauegram was drawn on the other side of the sample where a hypothetical back-reflected Laue pattern could be created. The virtual lauegram was created with the assumption that the incident beam was directed from the top to the point R_E_. In this case, the diffracted beam passes through the virtual lauegram in the spot r_d_. This spot can be transferred to the real lauegram, which is parallel to the incident beam. As a result of the transfer of the spot r_d_ from the virtual lauegram to the real lauegram, an extra spot is created. The position of the spot r_d_ was determined by inversion of the position of the spot r relative to the center of the lauegram.

The angles ε_EL_ and ε_ET_ between the CL^L^ and BL, as well as the CL^T^ and BL, respectively for the areas EL and ET, were determined on the basis of the shape of the micro-section II (Figure 1a). The ε_EL_ was 20° and the ε_ET_ was 40°. Afterwards, using the QLaue software, the Laue patterns recorded from the point R_E_ were rotated around the axis Z_L_ by using the angles ε_ET_ and ε_EL_, until the BL* of the lauegram lined up parallel to the CL^T^ and CL^L^ (lauegram II and III, Figure 5a). The reflex r (Laue pattern I), which was obtained from the (001)-type diffraction planes, changed location to r’ and r’’ on the rotated Laue patterns II and III, respectively. However, the transformed r_d_ spot was taken into account in further analysis because it is related to the direction of growing dendrites. As an example, the area ET of the upper sample was considered. In the cubic system, the directions of [001]-type are perpendicular to the crystal planes of (001)-type, which allowed us to define the inclination of the crystallographic direction [001] and the dendrites growth direction to the pressure and suction surfaces, meaning in relation to the walls of the mold. The primary arms growth direction may be defined by the position of the spot r_d_’ on the rotated lauegram II. Based on the rotated Laue pattern II and using the QLaue software, the rotation angles components α_M_ and α_N_ of the primary arms growth direction relative to the axes M and N, which are respectively perpendicular and parallel to the CL^T^ (X^T^), may be determined (Figure 5). When considering the blades obtained at a withdrawal rate of 5 mm/min, the angles were found to be α_M_ = 6.0° and α_N_ = 8.0° for the exemplary area ET (Figure 5a). It is important to note that for the angle δ_ET_, the component δ_ET_ of the rotation around the axis N of the primary arms growth may be calculated by using the equation γ_ET_ = α_N_ + δ_ET_ and in a more general case by the equation γ_ET_ = α_N_ ± δ_ET_. Additionally, the Laue pattern II allowed us to determine the angle β* using the equation β* = β − ε_ET_ (β* = −31 − 40 = −71°). The angle γ_EL_ may be determined similarly, using the rotated Laue pattern III.

As an example, for the upper sample, the angles of the surfaces inclination relative to the axis Z and rotation around the axis Z relative to the BL are as follows: for the area ET − $\delta_{\mathrm{ET}}=1.5{}^{\circ}$, $\varepsilon_{\mathrm{ET}}=40.0{}^{\circ}$, for the area EL − $\delta_{\mathrm{EL}}=11.0{}^{\circ}$, $\varepsilon_{\mathrm{EL}}=20.0{}^{\circ}$ (see Table 2).

Because the angle β* can take negative values (Laue pattern II, Figure 5), the absolute value of sin β* must be used in Equation (1). Therefore, the following Equation (2) should be used for the calculation of N:

(2) $$N=\frac{L*}{D}\tan\left(\alpha *\right)\times \left|\sin\left(\beta *\right)\right|$$

Taking into consideration the components α_M_ and α_N_ of the area ET and the fact that $\left(\alpha *\right)=\sqrt{\alpha_{M}^{2}+\gamma_{\mathrm{ET}}^{2}}=\sqrt{\alpha_{M}^{2}+{\left(\alpha_{N}\pm \delta_{\mathrm{ET}}\right)}^{2}}$, Equation (2) can be denoted into the following Equation (3):

(3) $$N=\frac{L*}{D}\times \tan\left[\sqrt{\alpha_{M}^{2}+{\left(\alpha_{N}\pm \delta_{\mathrm{ET}}\right)}^{2}}\right]\times \left|\sin\left(\beta \pm \varepsilon_{\mathrm{ET}}\right)\right|$$

The choice of the +/− sign was made on the basis of the localization of the image plate quadrant in which the spot r_d_’ is present.

As an example, calculations of the number of deflections N_ET_ for the area ET are presented below. In this case, there is the sign “−” in front of the δ_ET_ because the inclination δ_ET_ = 1.5° of the ET was consistent with the inclination of the dendrite relative to the axis N. The sign “−” also appears in front of the ε_ET_ because the rotation of the image plate was counterclockwise. Given the above, the Equation (3) takes the Equation (4).

(4) $$N_{\mathrm{ET}}=\frac{{L*}_{\mathrm{ET}}}{D_{\mathrm{ET}}}\times \tan\left[\sqrt{\alpha_{M}^{2}+{\left(\alpha_{N}-\delta_{\mathrm{ET}}\right)}^{2}}\right]\times \left|\sin\left(\beta -\varepsilon_{\mathrm{ET}}\right)\right|$$

Putting these values into Equation (4), we obtained:

(5) $$N_{\mathrm{ET}}=12.5\times \tan\left[\sqrt{6^{2}+{\left(8-1.5\right)}^{2}}\right]\times \left|\sin\left(\overset{-71{}^{\circ}}{\overset{︷}{-31{}^{\circ}-40{}^{\circ}}}\right)\right|=1.92\approx 2$$

Similar calculations can be made for the area EL of the upper sample. In this case, there is the sign “+” in front of the δ_EL_ because the inclination δ_EL_ = 11° (Figure 5a) of the leading edge and the surfaces’ EL was opposite to the inclination of the component α_N_ of the dendrite. The sign “+” also appears in front of the ε_EL_ because the rotation of the image plate was clockwise. Given the above, the equation for the N_EL_ takes the Equation (5).

(6) $$N_{\mathrm{EL}}=\frac{{L*}_{\mathrm{EL}}}{D_{\mathrm{EL}}}\times \tan\left[\sqrt{\alpha_{M}^{2}+{\left(\alpha_{N}+\delta_{\mathrm{EL}}\right)}^{2}}\right]\times \left|\sin\left(\beta +\varepsilon_{\mathrm{EL}}\right)\right|$$

Putting these values into Equation (5), the N_EL_ was obtained as follows:

(7) $$N_{\mathrm{EL}}=7.5\times \tan\left[\sqrt{9.5^{2}+{\left(3.5+11\right)}^{2}}\right]\times \left|\sin\left(\overset{-11}{\overset{︷}{-31{}^{\circ}+20{}^{\circ}}}\right)\right|=0.41\approx 0$$

The sinus and the tangent functions can take real numbers, therefore the calculated values were rounded to integers. The number N of the low-angle boundaries, which can be determined experimentally from the topograms obtained from the airfoil cross-section III (Figure 1a), is equal to the sum of the numbers of the low-angle boundaries created by deflection in the whole airfoil:

(8) $$N=N_{\mathrm{ST}}+N_{\mathrm{ET}}+N_{\mathrm{SL}}+N_{\mathrm{EL}}$$

In addition, two LABs from the root are usually inherited to the airfoil [28], which results in the equation:

(9) $$N=N_{\mathrm{ST}}+N_{\mathrm{ET}}+N_{\mathrm{SL}}+N_{\mathrm{EL}}+2$$

To verify the aforementioned mechanism of deflection, the data obtained experimentally in topograms were compared to the data calculated using Equation (7). Table 3 shows the experimental results and the calculations results of the number of LABs in the cross-section III for two series of blade casts obtained by using the Bridgman technique at the withdrawal rate of 2, 3, 4 and 5 mm/min and then heat treated.

The analysis of the data presented in Table 3 shows that there is good compatibility of the model and the experimental data. The differences between them are of the order of one LAB. However, for the airfoil of the series I of the blades cast at the withdrawal rate of 2 mm/min, the obtained topograms did not allow us to accurately determine the number of LABs. In the case of the subgrains marked as SG1, SG2, SG3 and SG4, one can state that the diffraction images of the dendrites (in the form of strips and spots) clearly formed groups with the same common crystallographic orientation. The other areas of the topograms were strongly and irregularly fragmented, therefore localization of subgrains and determination of the number of LABs was difficult. As a result, we could identify two more LABs (Table 3) in those areas, which are marked by two double red arrows in Figure 2a. The additional subgrain marked by the arrow with the envelope could be visualized in the topogram by using three contrast spots. In Figure 2b, all 5 marked LABs are clearly visible due to the lack of contrast bands that cross the entire width of the topogram. In Figure 2c, only the contrast band visualizing LAB no. 3 may be related to the macro-stress rather than to the misoriented subgrains; therefore, the number of boundaries counted may be one less than it otherwise would be. Analysis of the topogram presented in Figure 2d allowed us to indicate one additional LAB (marked by the red arrow). In the case of the topograms shown in Figure 2e–g, the determination of the LAB number was quite unambiguous. In the Figure 2h only the contrast band visualizing LAB no.6 could be questionable, therefore the counted number of boundaries may be one less than it otherwise would be because the contrast spot above LAB no.6 may belong to the group of dendrites located below LAB no.6. This is likely because the misorientation angle of LAB no.6 was close to 0.3°.

In the present study, the mechanism of dendrites deflection from the mold walls was verified regarding the heat-treated turbine rotor blades. The number of macroscopic LABs created on the cross-section of the blades airfoil near the tip was experimentally determined and compared to the LABs number calculated from the model based on the dendrites deflection mechanism.

Based on the Laue patterns and geometrical parameters of the airfoils, the number of the low-angle boundaries occurring at the upper part of the blades airfoil after heat treatment could be calculated. The number for the analyzed group of blades ranged from 5 to 9 (Table 3).

Due to the complex shape of the analyzed blades airfoil, which is similar in shape to the applied rotor blades, the aforementioned geometric assumptions based on the division into four flat areas were significantly simplified, but it is possible to verify this model. All the low-angle boundaries, which were formed during crystallization and inherited by the airfoil, appear near the blade tip. In the proposed model, to calculate the number of LABs in blades airfoils, it is necessary to experimentally determine the α* and β* angles used in Equation (1) and the values of the angles δ and ε describing the airfoil geometry. The values α* and β* can be determined in a non-destructive way by obtaining two Laue patterns directly from the pressure or suction surface, using, for example, the diffractometric method called Ω-scan and described in Ref. [39]. Determination of the number of LABs for blades airfoil allows for a non-destructive quality control process during blades production.

## 4. Conclusions

The dendrites deflection mechanism effectively describes the process of the low-angle boundaries creation in the airfoil of heat-treated rotor blades and allowed us to calculate the LABs number. It also confirms the proposed “deflection” mechanisms of the LABs creation in other thin-walled parts of single-crystalline casts. The dendrites deflection mechanism was verified for blades airfoils obtained by using directional crystallization in the direction [001] of the CMSX-4 superalloy at the withdrawal rates of 2 to 5 mm/min. The simplification of the geometric assumptions in the model could limit its use. However, the model can be the basis for more precise calculations. To increase the accuracy of the model, the complex shape of the blades should be taken into account, for example by using the finite element method. The proposed model is the basis for a non-destructive technique for determining the number of LABs in the blades airfoils, based only on two Laue patterns. Application of the method, without the need for cutting the blades and preparing metallographic sections, allows it to be used for quality control on the production lines.

## Figures and Tables

**Table 1:** The parameters of different X-ray topography methods and their applications.

The Method	X-ray Source	Incident Beam	Diffraction Geometry	Sample	Limit Resolution	Analyzed Crystals	
						Almost Perfect	Single-Crystalline Superalloys
Berg-Barrett	conventional	narrow parallel	reflection	small	arc seconds [33]	yes	yes
Lang	conventional	narrow parallel	transmission	small, thin	arc seconds [33]	yes	no
Applied in this study	conventional	wide divergent	reflection	large	arc minutes [34]	yes	yes
White beam	synchrotron	parallel	reflection/transmission	wide range	arc seconds [35]	yes	yes

**Table 2:** Characteristic geometric parameters of the SL, ST, EL and ET airfoil areas.

Airfoil Area	L* (mm)	D (mm)	L*/D	δ (°)	ε (°)
SL	15.0	3.7	4.05	7.5	45.0
ST	15.0	1.8	8.33	4.5	48.0
EL	15.0	2.0	7.50	11.0	20.0
ET	15.0	1.2	12.5	1.5	40.0

**Table 3:** The number of LABs defined by calculations and from X-ray topograms for airfoils of blades obtained in two series at the withdrawal rates of 2, 3, 4 and 5 mm/min.

	W (mm/min)	Number of Subgrains								Sum	Total (+2 from the Root)	Identified in Topograms
		ET		ST		EL		SL				
Series I	2	2.34	2	1.23	1	2.90	3	0.81	1	7	9	9
	3	1.20	1	0.16	0	2.21	2	1.12	1	4	6	5
	4	0.37	0	0.63	1	1.12	1	0.71	1	3	5	6
	5	1.92	2	1.22	1	0.41	0	0.03	0	3	5	5
Series II	2	1.47	2	1.7	2	0.18	0	0.20	0	3	6	6
	3	1.7	2	1.1	1	1.04	1	0.38	0	4	6	7
	4	2.06	2	1.36	1	1.97	2	0.38	0	5	7	7
	5	2.3	2	0.71	1	2.40	2	0.75	1	6	8	8

**Figure A1:**
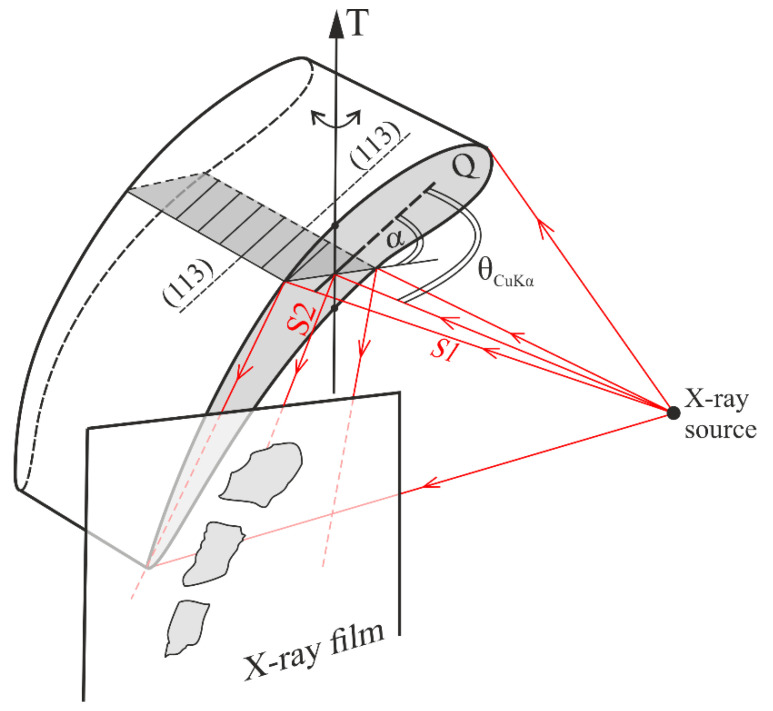
Diagram presenting the geometric arrangement of the X-ray source, sample and X-ray film for the X-ray topography method with the oscillation. S1—Incident divergent beam, S2—diffracted beam.

## Data Availability

Data sharing is not applicable to this article.

## Appendix A

The X-ray diffraction topography is based on the following principles. First, the upper sample is oriented using the Laue diffraction pattern obtained from its surface Q (marked by thin black downward arrows in Figure 1a). The sample is mounted in the holder so that the zone line defined by the reflections from planes (001) and (113) is arranged horizontally with the simultaneous vertical arrangement of the oscillation axis T of the sample and X-ray film (Figure A1). The sample is mounted in the center of the horizontal divergent incident beam (S1) and inclined at the Bragg angle $\theta_{\mathrm{CuK}\alpha }^{113}$ = 31° to the plane (113). The α, which is the angle between the sample surface and the plane (113), is determined from the Laue pattern. During exposure, the sample is oscillated about the T axis around the $\theta_{\mathrm{CuK}\alpha }^{113}$ angle within ±4°. The X-ray film is arranged parallel to the Q sample plane and to the T axis.

## Appendix B

The number N of dendrite deflections can be calculated for the area ET of the upper sample in the way presented below. Let’s calculate the number of dendrite arms deflections from the mold surface, based on the schemes from Figure 3c.

From Figure 3c and the assumption that h = 0 and b ≈ l, it follows that

(A1) $$N=\frac{L*}{l}$$

where L* is the height of the area ET.

From the triangle ΔNRT (Figure 3c) it follows that $l=\frac{m}{\cos\varphi }$, and from the triangle ΔNRM it follows that $m=\frac{D}{\sin\varphi }$.

The comparison of the above equations gives

(A2) $$l=\frac{D}{\sin\varphi \times \cos\varphi }$$

By putting Equation (A2) into Equation (A1), $N=\frac{L*}{D}\sin\varphi \times \cos\varphi$ is obtained, and the next the Equation (A3) is created

(A3) $$N=\frac{L*}{D}\sin\varphi \sqrt{1-\sin^{2}\varphi }$$

Considering the triangle ΔKRM in Figure 3b it can be determined that $\tan\left(\alpha *\right)=\frac{n}{b}$ and considering the triangle ΔKLM it can be determined that $\sin\left(\beta *\right)=\frac{D}{n}$.

The comparison of the above equations gives that the $\tan\left(\alpha *\right)\times \sin\left(\beta *\right)=\frac{D}{b}$. From Figure 3b,c it follows that $\frac{D}{b}=\tan\varphi$, so:

(A4) tan(α*)×sin(β*)=tanφ

Taking into account that $\tan\varphi =\frac{\sin\varphi }{\cos\varphi }$ and putting it into the Equation (A4) it follows that

(A5) $$\tan\varphi =\frac{\sin\varphi }{\sqrt{1-\sin^{2}\varphi }}=\tan\alpha *\times \sin\beta *$$

Treating the right side of the Equation (A5) as comparable to sin^2^φ, the equations (A6) are obtained as follow:

(A6) $$\sin^{2}\varphi =\frac{\tan^{2}\left(\alpha *\right)\times \sin^{2}\left(\beta *\right)}{1+\tan^{2}\left(\alpha *\right)\times \sin^{2}\left(\beta *\right)},\;\sin\varphi =\frac{\tan\left(\alpha *\right)\times \sin\left(\beta *\right)}{\sqrt{1-\tan^{2}\left(\alpha *\right)\times \sin^{2}\left(\beta *\right)}}$$

Putting the sin φ and sin^2^φ from Equation (A6) into Equation (A3), after simple transformation, Equation (A7) is obtained as follow:

(A7) $$N=\frac{L*}{D}\times \frac{\tan\alpha *\times \sin\beta *}{\left(1+\tan^{2}\alpha *\sin^{2}\beta *\right)}$$

Due to the low value of α* − tan^2^(α*) × sin^2^(β*) << 1.

Considering the above, the simplified equation, from Equation (A7), is given:

(A8) $$N=\frac{L*}{D}\times \tan\left(\alpha *\right)\times \sin\left(\beta *\right)$$
